# Supplementary material for: Impact of Bevacizumab on Visual Function, Tumor Size, and Toxicity in Pediatric Progressive Optic Pathway Glioma: A Retrospective Nationwide Multicentre Study
Source: Cancers (Basel). 2022 Dec 10;14(24):6087. doi: 10.3390/cancers14246087 (PMC9776082; doi:10.3390/cancers14246087)
Supplement: Supplementary file 1 [file cancers-14-06087-s001.zip › Supplementary tables-BVZ for OPG-V3-09122022.pdf]

Supplementary table S1: Individual characteristics of anatomic location, volumetric response and change in visual acuity/ visual field

Pt	MDC	Volume respons (RAPNO)	BCVA OD Start[1]	VF start OD	BCVA OS start[1]	VF start OS	Change BCVA OD[1]	Change VF OD	Change BCVA OS[1]	Change VF OS
**1**	2A-3	PD	NDA	NDA	NDA	NDA	NDA	NDA	NDA	NDA
**2**	2B-3(L>R)	MR	0,4	Relative partial superior arcuate defect & absolute inferior altitudinal defect	0,2	Absolute complete temporal hemianopia and partial quadrantanopia inferior nasal	-0,13	↑	-0,15	↑
**3**	2-3R	SD	3,0	NDA	0,2	Absolute complete temporal hemianopia and partial quadrantanopia inferior nasal	0	NDA	0,17	↓
**4**	2A	PR	0,1	Central scotoma	0	Central scotoma	-0.06	↑	0	↑
**5**	2-3L	MR	0,4	Absolute complete temporal hemianopia & absolute partial nasal inferior quadrantanopia	1,8	NDA	-0,36	↑	0	NDA
**6**	1A-2B(L)	SD	0	No defect	1,5	NDA	0	↓	1,2	NDA
**7**	1C-2A	SD	0	Combined absolute/ relative concentric defects	0,1	Combined absolute/ relative concentric defects	0,1	↑	NDA	↑
**8**	2a-3(R+L)	PD	NDA	NDA	NDA	NDA	NDA	NDA	NDA	NDA
**9**	1B(L>R)-2A-3(L>R)-4B (L)	PR	NDA	NDA	NDA	NDA	NDA	NDA	NDA	NDA
**10**	2A	PR	1,8	NDA	3,0	NDA	0	NDA	0	NDA
**11**	1C(R> L, 2A	SD	3,0	NDA	3,0	NDA	0	NDA	0	NDA
**12**	2A3B	SD	NDA	NDA	NDA	NDA	NDA	NDA	NDA	NDA
**13**	1B(L>R)-2A-3	SD	0,3	NDA	3,0	NDA	-0,25	NDA	0	NDA
**14**	2A-3B(R)	PR	2,8	NDA	1,1	Absolute concentric contraction	0	NDA	0	↑

**15**	1A-1C(L), 2(A>R)	SD	-0.08	NDA	3,0	NDA	0	NDA	NDA	NDA
**16**	1C-2A-3A	MR	3,0	NDA	3,0	NDA	0	NDA	0	NDA
**17**	2A-3B	MR	1,6	Concentric contraction	2,7	Concentric contraction	-0,3	↑	-0,7	Stable
**18**	2A	SD	1	Absolute complete temporal hemianopia, partial nasal quadrantanopia	2,7	NDA	0,3	↑	0,1	NDA
**19**	2A-3-4B (L)	SD	1,3	Absolute partial quadrantanopia	2	Temporal rest <20 degrees	-0,6	↑	-0,3	↑
**20**	1B-2A-3-4	No data	0,2	NDA	0,3	NDA	0,12	NDA	0,02	NDA
**21**	1A(L)-2B(R)-3B (L>R)	SD	0	Enlarged blind spot	0,5	No abnormalities	0,03	Stable	0	Stable
**22**	1B-2A-3 (L>R)	MR	0,9	NDA	3,0	NDA	-0.22	NDA	0	NDA
**23**	2A-3B	SD	0,8	NDA	1,3	NDA	-0,01	NDA	0	NDA
**24**	2A-3	PR	3,0	NDA	3,0	NDA	0	NDA	0	NDA
**25**	2A	PR	NDA	Absolute complete temporal hemianopia	NDA	NDA	NDA	Stable	NDA	NDA
**26**	1A(R)-2A	SD	0,2	Incomplete absolute temporal hemianopia	0	Partial relative superior bow scotoma	0,01	↑	-0,04	Shift [3]
**27**	2A-3A(L)	SD	NDA	NDA	NDA	NDA	NDA	NDA	NDA	NDA
**28**	2A-3(L>R)	SD	3,0	NDA	1	NDA	0	NDA	0,04	NDA
**29**	2A-3B	MR	0,4	Absolute incomplete temporal hemianopia	0,2	Absolute incomplete temporal hemianopia	-0,4	↑	-0,19	↑
**30**	2A-3B	PD	0,1	Absolute incomplete temporal hemianopia	0,4	Absolute incomplete nasal hemianopia	0	↑	-0,04	↑
**31**	2A-3B(R)	SD	NDA	NDA	NDA	NDA	NDA	NDA	NDA	NDA
**32**	1B 2A 3 4(R>L)	PR	2,7	NDA	3,0	NDA	0,1	NDA	0	NDA
**33[2]**	1B L>R 2A R>L 3A-4	MR	0.02	Absolute cecocentral scotoma	0.05	Absolute cecocentral scotoma	-0.09	↑	-0.12	↑

Ad [1]: BCVA in LogMAR
Ad [2]: Ongoing BVZ treatment, evaluation 6 months after start BVZ
Ad [3]: VF shift: VF loss in one quadrant, which on subsequent VF evaluation changes to VF loss in a different quadrant

Abbreviation: BCVA: Best Corrected Visual Acuity, L: left, MDC: Modified Dodge classification (stage 1: optic nerve(s),stage 2: chiasm, stage 3: optic tract, stage 4: posterior optic tract), MNP: measurements not possible due to diffuse location of OPG with minor chiasmal involvement, MR: minor response, NDA: no data available, OD: Oculus Dexter = right eye, OS: Oculus Sinister= left eye, PD: progressive disease, R: right, SD: stable disease, VF: Visual Field, ↑: improvement, ↓: decrease

Supplementary Table S2: Type of therapy applied per treatment phase for OPG.

Type of therapy per phase	Nr of patients (%)
**SAT in episode 1**	**33**
Carboplatin/vincristin	30 (90.9)
Carboplatin/vincristin/etoposide	1 (3.0)
Vinblastine	1 (3.0)
Bevacizumab	1 (3.0)
**SAT in episode 2**	**32**
Vinblastine	17 (53.1)
Vinblastine/carboplatin	2 (6.2)
Bevacizumab	13 (40.6)
**SAT in phase 3**	**22**
Temodal	3 (13.6)
Vinblastine	2 (9.1)
Vinorelbin	1 (4.5)
Bevacizumab	16 (72.7)
**SAT in phase 4**	**3**
Bevacizumab	3 (100.0)
**Previous neurosurgical resection prior to BVZ**	9
1x resection	8 (24.4)
4x resection	1 (3.0)
**Previous radiotherapy**	1
1x	1 (3.0)

Abbreviations: SAT: systemic antitumor therapy.

Supplementary table S3: Individual dosage and treatment schedule of bevacizumab, irinotecan and vinblastine.

Pt	Nr of doses: BVZ	Nr of doses: IRI	Nr of doses: VBL	Interval BVZ/ IRI	Interval VBL	Dosage BVZ	Dosage IRI	Dosage VBL
**1**	26	26	-	1x 2 weeks		10 mg/kg	125mg/m^2	-
**2**	45	26	-	1x 2 weeks		10 mg/kg	125mg/m^2	-
**3**	9	9	-	1x 2 weeks		10 mg/kg	125mg/m^2	-
**4**	26	12	-	1x 2 weeks		10 mg/kg	125mg/m^2	-
**5**	11	-	11	1x 2 weeks	weekly	10 mg/kg	-	1.5 mg/m^2
**6**	25	25	-	1x 2 weeks		10 mg/kg	125mg/m^2	-
**7**	9	9	-	1x 2 weeks		10 mg/kg	125mg/m^2	-
**8**	12	12	-	1x 2 weeks		10 mg/kg	125mg/m^2	-
**9**	52	52	-	1x 2 weeks		10 mg/kg	125mg/m^2	-
**10**	24	14	-	1x 2 weeks		10 mg/kg	125mg/m^2	-
**11**	12	6	-	1x 2 weeks	weekly	10 mg/kg	-	3 mg/m^2
**12**	16	16	-	1x 2 weeks		10 mg/kg	125mg/m^2	-
**13**	19	19	-	1x 2 weeks		10 mg/kg	125mg/m^2	-
**14**	26	-	-	1x 2 weeks		10 mg/kg	-	-
**15**	30	14	-	1x 2 weeks		10 mg/kg	125mg/m^2	-
**16**	65	50	-	41 doses 1x/ 2 weeks<br>15 doses 1x/ 3 weeks<br>9 doses, 1x/ 4 weeks		41x at 10 mg/kg<br>24 at 9 mg/kg	125mg/m^2	-
**17**	26	14	-	1x 2 weeks		10 mg/kg	125mg/m^2	-
**18**	15	15	-	1x 2 weeks		10 mg/kg	125mg/m^2	-
**19**	18	18	-	1x 2 weeks		10 mg/kg	125mg/m^2	-
**20**	26	21	-	1x 2 weeks		10 mg/kg	125mg/m^2	-
**21**	30	13	-	1x 2 weeks		10 mg/kg	125mg/m^2	-
**22**	26	26	-	1x 2 weeks		10 mg/kg	125mg/m^2	-
**23**	22	10	-	1x 2 weeks		10 mg/kg	125mg/m^2	-
**24**	31	31	-	1x 2 weeks		10 mg/kg	125mg/m^2	-
**25**	26	-	9	1x 2 weeks	weekly	10 mg/kg	-	3 mg/m^2
**26**	21	21	-	1x 2 weeks		10 mg/kg	125mg/m^2	-
**27**	19	-	38	1x 2 weeks	weekly	10 mg/kg	-	3 mg/m^2
**28**	89	28	-	1x 2 weeks		10 mg/kg	125mg/m^2	-
**29**	34	34	-	1x 2 weeks		10 mg/kg	125mg/m^2	-
**30**	4	4	-	1x 2 weeks		10 mg/kg	125mg/m^2	-
**31**	16	-	32	1x 2 weeks	weekly	10 mg/kg	-	3 mg/m^2
**32**	33	-	66	1x 2 weeks	weekly	10 mg/kg	-	3 mg/m^2
**33**	39	13	-	1x 2 weeks		10 mg/kg	125mg/m^2	-

Abbreviations: BVZ: bevacizumab, IRI: irinotecan, VBL: vinblastine
